# On Going out of Insurance

**Published:** 1914-01-24

**Authors:** 


### NATIONAL INSURANCE ACT DEVELOPMENTS.

### On Going Out of Insurance.

In the exercise of their duty of making clear the provisions of the National Insurance Acts, the Commissioners have recently seen fit to issue a circular (A.S. 113) to all approved societies, dealing with the very important question as to the circumstances in which an "insured person" ceases to be eligible for the benefits of the Acts as an employed contributor.

Few people seem to realise how important a matter this is, and we therefore take this opportunity of discussing the position for the benefit of insured persons.

When a contributor ceases to be employed as defined by the Acts, such contributor, generally speaking, is at once deprived of the ordinary benefits of the Acts; if, therefore, no trouble is taken when a change of occupation occurs to inquire whether this change causes the contributor to cease to be insured, the latter may find himself in a somewhat invidious position. It must be clearly understood that ineligibility to be insured disentitles an insured person both to pay contributions and to receive benefits, and should he in spite of this continue to keep up his payments, the approved society to which he belongs would be in a position to refuse to pay a claim. It may be remarked in this connection that before paying benefits approved societies necessarily require evidence that the claimant is entitled to the benefit claimed.

The wording of the Act on the general question under consideration is typically ambiguous, and the Commissioners indicate in the opening paragraph of their remarks that the circular is an effort to clear away the misunderstanding that appears to exist both in the minds of insured persons and of those who control the operations of approved societies.

In order to understand how the confusion has arisen it should be borne in mind that an insured person may cease to be employed within the meaning of the Acts in four main ways, some of which are temporary while others are permanent in their effect: (*a*) by being rendered incapable of work through sickness or disablement; (*b*) by being unable to obtain employment owing to a scarcity of demand for labour; (*c*) by changing his or her occupation to one which is not employment within the meaning of the Act; and (*d*) by permanent retirement.

In the case of incapacity for work on account of illness, no difficulty should arise, as if this is the only reason for the unemployment of an insured person, the latter in no way ceases to be insured: to quote the words of the Commissioners: "Any other interpretation would clearly defeat the whole purpose of the Act. No question therefore of a person ceasing to be insured can possibly arise unless and until he is in a fit state of health to work, and this general principle would apply even though there might be reason to suppose that the person would not resume work after his recovery. For example, in the case of a woman who ceases to be employed by reason of her being rendered incapable of work shortly before her confinement, the question of her ceasing to be insured should not be raised until she has recovered and is again in a fit state of health to resume work."

It is thus with regard to (*b*), (*c*), and (*d*) above that the chief difficulties have arisen.

### Temporary Unemployment.

With respect to (*b*) the matter is at present governed by the fourth paragraph of Section 79 of the principal Act. This paragraph lays down the rule that a person whose normal occupation is employment within the meaning of the Act is to be deemed to continue to be an employed contributor, in spite of the fact that he may become temporarily unemployed. If, however, such period of unemployment extends beyond twelve months he cannot continue to be an employed contributor unless the approved society of which he is a member is satisfied that his unemployment is due to inability to obtain employment and not to any change in his normal occupation.

From the wording of the paragraph it would seem that after the lapse of this period of twelve months the onus of proving that he is entitled to remain an insured person rests with the member of the society, and if he does not take steps in this direction he apparently ceases forthwith to be insured.

Care must be taken to distinguish between "ceasing to be a member of an approved society" and "ceasing to be eligible for benefits." Suspension of a member of an approved society from the benefits of the Acts through non-payment of arrears or from any other cause, does not in itself deprive such a member of his membership; he continues to be an insured person although not entitled to receive benefits.

Thus, while a contributor "ceasing to be an insured person" is necessarily rendered ineligible for benefits it does not follow that the converse holds—namely, that a person who cannot claim benefits is not an insured person; the distinction is important and should be kept in mind.

The effect of the above-explained paragraph of Section 79 is "to give to . . . employed contributors a period of grace of twelve months, during which, while for the time being unemployed, they are entitled to continue to pay contributions at the employed rate and to receive benefits subject to the usual conditions." This twelve months' grace is, however, only applicable provided the unemployment is due to inability to obtain work, and it is largely with regard to this that confusion has arisen.

If during the first year after ceasing to be employed within the meaning of the Acts there is a definite and apparently permanent change in the status of a contributor, the Commissioners instruct that he must be provisionally treated as having gone out of insurance, and the society must notify the Insurance Committee to that effect. If, however, he should return to employment before the end of the year he may be reinstated as an employed contributor, as though he had remained so during the period in which his status was changed. If he does not so return " the lapse from insurance is made absolute unless the society decides at the end of the year that the reason for the member not being employed is inability to obtain employment and not a change in his normal occupation."

Permanent Change of Employment.

It is interesting and important to note that, within the year of grace, approved societies are only to treat employed contributors as having gone out of insurance provided that the change of status which is the basis of such treatment is both definite and apparently permanent. It seems that approved societies will have to decide, before taking action, upon the permanence or otherwise of any such change, and this in certain circumstances may be difficult to do. Should the change of status be permanent the contributor concerned may, of course, if qualified, elect to continue insurance as a voluntary contributor.

The following examples are given by the Commissioners of a definite change of status such as referred to above: (1) a person leaving the United Kingdom with no definite intention of returning within a year; (2) a person employed otherwise than by manual labour whose rate of remuneration is raised definitely above £160 a year; (3) a person who enters into an employment specifically excepted from insurance under the provisions of the Acts; (4) a person who enters upon some other occupation, apparently of a permanent character, which is not employment within the meaning of the Acts.

It may be remarked that a person who ceases to be an employed contributor on account of the fact that his income has risen to over £160 a year is not at present entitled to the privilege of voluntarily continuing his insurance, as the Act requires that for this purpose he must have been an insured person for a period of at least five years.

If a person passing out of insurance as an employed contributor in any of the above or similar ways, remains so for a period exceeding one year, and does not elect to continue insurance voluntarily, it would appear that if subsequently he again becomes employed in such a way as to bring him within the scope of the Acts, he will on his re-entry have to pay a higher contribution or suffer a reduction in benefits according to the scale applicable to his then advanced age.

Married Women.

It may now be profitable to consider the application of the preceding remarks to married women.

Married women employed contributors who were not insured before marriage are subject to precisely the same regulations as have been enunciated above. " A member of this class who ceases work in order to devote herself to domestic duties will be treated in the same way as an employed contributor who ceases to be employed and takes up some other occupation which is not employment within the meaning of the Acts."

Women who are insured persons and unmarried present, however, a case of considerable difficulty. Special provision is made for such insured persons, in the event of their contracting marriage, by Section 44 of the National Insurance Act, 1911.

Should a woman employed contributor continue to be employed after marriage, she is in no way suspended from the ordinary benefits of the Acts unless she ceases to be employed. " When, however, a woman who was insured before marriage has definitely established her position as a person whose normal occupation after marriage is employment within the meaning of the Acts . . . she then comes into the same position as a married woman employed contributor who was not insured before marriage "; that is to say, she is subject to the ordinary regulations regarding insured persons ceasing to be employed.

Married women who were before marriage insured persons and have exercised their right to become special voluntary contributors are also given consideration by the Commissioners. If such persons subsequently become employed in a manner that will bring them within the operation of the Acts, two courses are open to them; either to become ordinary employed contributors or to obtain a certificate of exemption from insurance.

The adoption of the former course has in many respects distinct disadvantages. For example, if such married women become employed contributors they do not on again ceasing to be employed revert to the position of special voluntary contributors, but are subject to the same conditions as if they had not been insured before marriage.

There are, of course, some advantages to be gained by claiming the certificate of exemption from insurance. Persons so exempted are entitled under the provisions of the Act of 1913 (Section 9) to medical and sanatorium benefits as though they were members of approved societies. That is to say, although they do not pay any contributions themselves—only the employer's 3d. being payable—they are entitled to receive the medical treatment and attendance provided by the Acts. And although medical treatment under the panel system is not all that can be desired, it is at least worth something. Furthermore, it would appear that if a woman who has been a special voluntary contributor exercises her right when she resumes employment to claim a certificate of exemption from insurance on the ground that she is mainly supported by her husband, she does not forfeit her right to continue or resume her status as a special voluntary contributor. In other words, she can still claim medical benefit and the benefits during sickness, otherwise than on confinement, of 5s. a week for the first thirteen weeks and 3s. a week during the second thirteen weeks, together with disablement benefit of 3s. a week. If, therefore, a woman who has been a special voluntary contributor desires to preserve her right to remain in that class she should not re-enter as an employed contributor but obtain a certificate of exemption.

